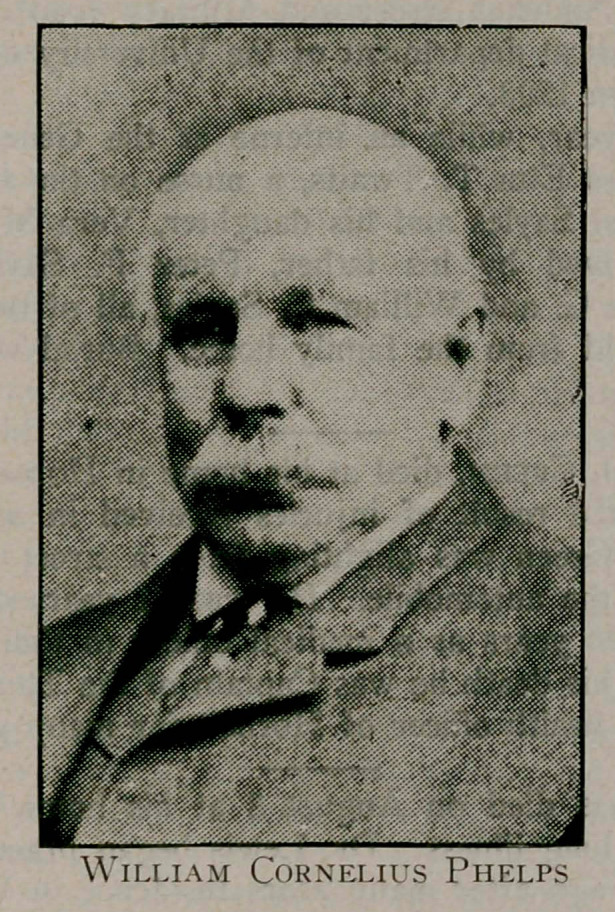# William Cornelius Phelps, M.D.

**Published:** 1911-09

**Authors:** 


### OBITUARY

WILLIAM CORNELIUS PHELPS, M.D., was born at Vernal Corners, N. Y., August 31, 1844, of Israel Root Phelps and Eliza Van-Allen Phelps. He died at 146 Allen St., Buffalo, August 27,

WILLIAM CORNELIUS PHELPS

1911, after nearly a year's illness dating from a fall by which his patella was fractured.

Dr. Phelps, after graduating from Middlebury Academy, registered for the study of medicine with the late Dr. John D. Hill of Buffalo, and received the degree of M.D. from the University of Buffalo in 1866.

He held the following positions of honor and professional activity: Surgeon of 74th Regiment, Police Surgeon, in the early '70's; twice Health Physician of Buffalo, the second time for the years 1882 and 1883; until two years ago, Demonstrator of Anatomy, University of Buffalo; Surgeon, Buffalo General and Erie County Hospitals; President of Staff of the latter.

Dr. Phelps practised medicine for forty-five years in Buffalo, being of the best type of the general practitioner and surgeon. He was a modest, kindly, substantial gentleman, conservative in his methods, advanced in his knowledge, never advancing his attainments for selfish ends. Many of the old boys who dissected under his guidance will join us in a tribute to his ability as a teacher and his worth as a friend.

## Figures and Tables

**Figure f1:**